# Engineered interfaces between perovskite La_2/3x_Li_3x_TiO_3_ electrolyte and Li metal for solid-state batteries

**DOI:** 10.3389/fchem.2022.966274

**Published:** 2022-08-10

**Authors:** Shuo Yan, Hilal Al-Salih, Chae-Ho Yim, Ali Merati, Elena A. Baranova, Arnaud Weck, Yaser Abu-Lebdeh

**Affiliations:** ^1^ Department of Chemical and Biological Engineering, Centre for Catalysis Research and Innovation (CCRI), University of Ottawa, Ottawa, ON, Canada; ^2^ National Research Council of Canada, Ottawa, ON, Canada; ^3^ Department of Mechanical Engineering, University of Ottawa, Ottawa, ON, Canada

**Keywords:** solid-state electrolyte, perovskite LLTO, highly dense microstructure, interfacial modifcation, conductive ceramic, lithium metal battery

## Abstract

Perovskite La_2/3x_Li_3x_TiO_3_ (LLTO) materials are promising solid-state electrolytes for lithium metal batteries (LMBs) due to their intrinsic fire-resistance, high bulk ionic conductivity, and wide electrochemical window. However, their commercialization is hampered by high interfacial resistance, dendrite formation, and instability against Li metal. To address these challenges, we first prepared highly dense LLTO pellets with enhanced microstructure and high bulk ionic conductivity of $2.1\times 10^{-4}$ S cm^−1^ at room temperature. Then, the LLTO pellets were coated with three polymer-based interfacial layers, including pure (polyethylene oxide) (PEO), dry polymer electrolyte of PEO-LITFSI (lithium bis (trifluoromethanesulfonyl) imide) (PL), and gel PEO-LiTFSI-SN (succinonitrile) (PLS). It is found that each layer has impacted the interface differently; the soft PLS gel layer significantly reduced the total resistance of LLTO to a low value of 84.88 Ω cm^−2^. Interestingly, PLS layer has shown excellent ionic conductivity but performs inferior in symmetric Li cells. On the other hand, the PL layer significantly reduces lithium nucleation overpotential and shows a stable voltage profile after 20 cycles without any sign of Li dendrite formation. This work demonstrates that LLTO electrolytes with denser microstructure could reduce the interfacial resistance and when combined with polymeric interfaces show improved chemical stability against Li metal.

## Introduction

The urgent demand for clean and renewable power sources is greatly stimulated by the constant rise of global GHG emissions (Hu et al., 2019). Solid-state lithium metal batteries (LMBs) are promising alternative energy sources with increased energy/power density that can significantly reduce GHG emissions in various high-polluting industrial sectors, such as transportation. Lithium (Li) metal has an ultrahigh theoretical capacity (3,860 mAh g^−1^) and the lowest electrochemical potential (–3.04 V vs. the standard hydrogen electrode) of all metals coupled with very low density (0.534 g/cm^3^) (Tarascon and Armand, 2001; Xu et al., 2014; Chen et al., 2019). However, lithium dendrite formation and propagation in traditional LMBs with flammable liquid-state organic electrolytes pose safety and electrochemical performance issues (Lv et al., 2019). Moreover, solid-state electrolytes permit reliable safety for LMBs due to their non-flammable, solid feature, and the wider electrochemical window (>5 V) (Lotsch and Maier, 2017; Wang et al., 2020).

Among reported solid-state electrolyte materials, perovskite-type (ABO_3_) LLTO exhibits high bulk ionic conductivity, good stability in a dry or humid atmosphere and a wide operating temperature range (Wang et al., 2019; Yan et al., 2021). However, the low sinterability (long period of sintering at a high temperature over 1,000°C) leads to significant Li loss, further resulting in a decreased lower total ionic conductivity. Simply modifying the sintering conditions risks reducing the density of electrolytes. To maintain good sinterability and density, scholars typically add additional Li [i.e., excess of pristine powders (Jonson and McGinn, 2018) and low-melting points salts (Le et al., 2013; Li Y. et al., 2018a)] during solid-state processing. However, the produced second phase may increase the grain boundary resistance.

In addition, the resistive and inhomogeneous interfacial contact against Li metal results in high interfacial resistance in the range of 10^2^–10^3^ Ω cm^−2^ (Fu et al., 2017; Shen et al., 2018; Xu et al., 2018). High interfacial resistance due to poor contact results in a large overpotential during the charge and discharge cycling process. Various strategies of surface treatments on ceramic electrolytes have been investigated to improve the interfacial contact. Table 1 presents selected interfaces on ceramic electrolytes and performance in symmetric Li cells.

**TABLE 1:** Summary of selected interfaces on ceramic electrolytes and performance in symmetric lithium cells.

Interface	Ceramic SSEs	Interface resistance (ohm cm^−2^)	Stable potential (mV)
Au Tsai et al. (2016)	Ta-doped Li_7_La_3_Zr_2_O_12_ (LLZTO)	Reduced from 1,500 to 380	∼22 at 0.5 mA cm^−2^
20 nm germanium (Ge) Luo et al. (2017)	Li_6.85_La_2.9_Ca_0.1_Zr_1.75_Nb_0.25_O_12_ (LLZO)	Reduced from 900 to 115	∼25 at 0.05 mA cm^−2^
Al_2_O_3_ Han et al. (2017)	Li_7_La_2.75_Ca_0.25_Zr_1.75_Nb_0.25_O_12_ (LLCZN)	Reduced from 1710 to 1	22 at 0.2 mA cm^−2^
ZnO Wang et al. (2017)	Li_6.75_La_2.75_Ca_0.25_Zr_1.75_Nb_0.75_O_12_	20	6.5 at 0.1 mA cm^−2^
Graphite Shao et al. (2018)	Li_5.9_Al_0.2_La_3_Zr_1.75_W_0.25_O_12_ (LALZWO)	Reduced from 1,350 to 105	6 at 50 μ A cm^−2^
PEO Jiang et al. (2020)	Li_0.34_La_0.56_TiO_3_ (LLTO)	549	100 at 0.1 mA cm^−2^
PEO/LiTFSI (O/Li mole ratio of 8:1) Chi et al. (2019)	Li_6.4_La_2_Zr_1.4_Ta_0.6_O_12_ (LLZTO)	Data is no available	50 at 0.1 mA cm^−2^
Cross-linked poly (ethylene glycol) methyl ether acrylate-LiTFSI-Al_2_O_3_(CPAMEA) Zhou et al., 2016	Li_1.3_Al_0.3_Ti_1.7_(PO_4_)_3_ (LAGP)	Data is not available	500
PAN/10 wt% LiClO_4_ Yin et al., (2020)	Li-ion-conducting glass ceramic	Data is not available	150 at 0.3 μ A cm^−2^
PVDF-HFP Liu et al. (2017)	Li_7_La_2.75_Ca_0.25_Zr_1.75_Nb_0.25_O_12_ (LLCZN)	Reduced from 1,400 to 214	125 at 125 μ A cm^−2^
PLS gel membrane (this work)	La_0.57_Li_0.29_TiO_3_ (LLTO)	Reduced to 84.88 for pellet	7.25 at 0.04 mA cm^−2^

One approach is to introduce another metallic layer between the Li metal and ceramic electrolytes such as gold (Au) (Tsai et al., 2016), germanium (Ge) (Luo et al., 2017), etc. Alternatively, Fu et al. (Fu et al., 2017) applied an aluminum metal coating on garnet and reduced the interfacial resistance by more than one order of magnitude. A metallic layer tends to alloy with Li metal, which could improve the wettability of ceramic electrolytes though they are still solid and hence, cannot ensure impeccable surface contact with solid electrolytes. To further enhance the contact against electrodes, metal oxides consisting of Al_2_O_3_ (Han et al., 2017), ZnO (Wang et al., 2017), and graphite (Shao et al., 2018) are widely used to modify the surface of electrolytes. Han et al. (2017) deposited atomic-layer of Al_2_O_3_ on garnet electrolyte, allowing the interfacial resistance to be reduced to only 1 Ω cm^−2^. However, the above-mentioned metal oxides are generally deposited by complicated and expensive methods. The resistance between hard ceramic grains within the electrolyte is still large. Compared with metal or metal oxides, utilizing soft polymers (i.e., polyethylene oxide) (PEO) (Zhou et al., 2016; Chi et al., 2019; Jiang et al., 2020), polyacrylonitrile (PAN) (Yin et al., 2020), polyvinylidene fluoride (PVDF)-HFP (Liu et al., 2017), etc.) as interfacial layers increases ionic conductivity and enhances the contact among neighboring grains by filling the voids and grain boundary regions (Liu et al., 2018; Cheng et al., 2019). Specifically, PEO has been widely used for composite electrolytes to provide flexibility but suffers low room temperature conductivity (10^−6^∼10^−8^ S cm^−1^) partly due to its high degree of crystallinity below its melting point (∼60°C). The addition of Li salts such as lithium bis (trifluoromethanesulfonyl) imide (LiTFSI), tends to reduce PEO crystallinity and enhance conductivity by facilitating lithium-ion transport. Al-Salih et al. (Al-Salih et al., 2020) published on polymer-rich electrolytes (the mass ratio of PEO:LiTFSI is 70%:30%) with ionic conductivity of >10^−3^ S cm^−1^ at 55°C. As an effective ionizer, succinonitrile’s (SN) high dielectric nature can separate Li ions from LiTFSI and further boost ionic conductivity. Observed room temperature ionic conductivity could reach the magnitude of 10^−3^ S cm^−1^ by optimizing the mass ratio to 35% PEO:30% LiTFSI:35% SN (Echeverri et al., 2012). They have also shown successful cycling in oxide-based cathodes up to 4.1 V compared to the conventional, phosphate-based cathode limited by PEO stability above 3.7 V.

To date, only a few articles have proposed microstructural and interfacial modifications to perovskite-based electrolytes. The main problem with perovskite-based electrolytes is that Ti^4+^ is chemically unstable in the presence of Li-metal. LLTO reacts rapidly with Li accompanying with its color shift from white to black (Yan et al., 2018). Galvez-Aran et al. (Galvez-Aranda and Seminario, 2020) further confirmed that Ti reduction occurs at the LLTO/Li-metal anode interface. The reaction rate increases as the applied external electric field increases, indicating that LLTO is electrochemically unstable with Li. As evidenced by X-ray photoelectron spectroscopy (XPS) studies, Ti^4+^ in LLTO reduces into lower valence Ti species (e.g., Ti^3+^, Ti^2+^, and Ti^0^)(Wenzel et al., 2015; Liu et al., 2017). Furthermore, Ti reduction is associated with the production of oxygen vacancies, making the interface electrically conductive and unsuitable as an electrolyte for LMBs.(Liu et al., 2017). Thus, it is essential to introduce protective layers for LLTO to avoid direct contact with Li and lower the interfacial resistance of Li^+^ transportation. (Ji et al., 2020) (Jiang et al., 2020). applied PEO films between LLTO and electrodes in a cell of Li | PEO | LLTO | PEO | (LiFePO_4_) LFP that exhibited good electrochemical performance. The initial discharge capacity reached 145 mAh g^−1^ at a current density of 0.1C and capacity retention was 86.2% after 50 cycles.

In this work, we have first prepared dense LLTO electrolyte materials with improved microstructure and high bulk ionic conductivity by mixing and optimizing the weight ratios between granular and milled LLTO powders at different sintering temperatures. We have then identified optimal interfacial layers of PEO-based gel membranes for LLTO electrolytes, including 100 wt% PEO (P), 70 wt% PEO/30 wt% LiTFSI (PL), and 35 wt% PEO/30 wt% LiTFSI/35 wt% SN (PLS). The optimal interfacial layer for LLTO is expected to provide efficient protection against Li metal and reserve good ionic conductivity. Electrochemical Impedance spectroscopy was performed to evaluate the conductivity of pristine and coated LLTO pellets. Symmetric Li cells and Ohm’s law were used to explore and analyze the possible mechanism of coated LLTO electrolytes for the improved stability with Li metal.

## Materials and methods

### Electrolyte fabrication

Pristine La_0.57_Li_0.29_TiO_3_ (TOHO TITANIUM, Co., Ltd.) powders including granular (G-LLTO) and milled (M-LLTO) type were stored under inert conditions in an argon-filled glovebox. To prepare mixed LLTO powders, G-LLTO and M-LLTO with optimized weight ratio of 70:30 were dispersed in 2-propanol to acquire a homogenous suspension. After vigorous stirring at 2000 rpm for 30 min (Thinky mixer), the mixture was dried in a 70°C oven overnight to remove the solvent.

The LLTO powder was then uniaxially cold-pressed followed by sintering at high temperature. The mixed powder was molded in a stainless-steel die (15.6 mm in diameter) and then pressed with a pressure of 200 MPa for 4 min. The cold-pressed pellet was fully covered with mother powder and then was sintered at 1,170°C for 12 h in air. The mother powder was added to compensate for any lithium loss at the high-temperature sintering process ^29^. The heating and cooling rate were 10°C/min and 2°C/min, respectively. The sintered electrolyte was cut into about 0.7 mm thick slices by a low-speed diamond saw (MTI Co., Ltd.) and then stored in an argon-filled glovebox.

### Interfacial modifications

Poly (ethylene oxide) (PEO, average molecular weight of 600,000) was dispersed in acetonitrile to acquire a pure PEO (P) solution. PEO and lithium bis (trifluoromethanesulfonyl) imide (LiTFSI) with a weight ratio of 70:30 was mixed in acetonitrile to get PEO-LiTFSI (PL) solution. An optimal weight ratio among PEO, LiTFSI, and succinonitrile (SN) of 35:30:35 in acetonitrile was used to make the gel membrane (PLS) solution. Each solution was dropped two times on both sides of LLTO pellets. The coated pellets were dried in air at room temperature to remove the solvent.

### Symmetric Li cells assembly

Symmetric Li cells of LLTO pellets with and without coating were fabricated in an argon-filled glovebox to investigate the process of Li plating/stripping and evaluate its long-term cycling stability. Two Li foils were sandwiched on both sides of coated LLTO in a 2325-coin cell.

### Material characterization

The morphology of the pristine powder and prepared pellets were observed by scanning electron microscopy (Zeiss Gemini SEM 500) and energy dispersive X-ray spectrometry (EDS, Bruker). The crystalline phase was confirmed by powder X-ray diffractometry (PXRD, Rigaku, Ultima IV) with a copper source and one diffracted beam monochromator, operating at 40 kV and 44 mA. The pellets were scanned in the 2 θ range from 10 to 60° with a scan rate of 2°/min. X-ray photoelectron spectroscopy (XPS) analyses were carried out using an Axis Ultra DLD spectrometer (Kratos Analytical, Manchester, UK) with monochromatized Al K α X-rays.

### Electrochemical characterization

The resistance of pellets was measured via electrochemical impedance spectroscopy (EIS) by impedance/gain-phase analyzer (Solartron, SI 1,260) with a frequency ranging from 1 MHz to 0.01 Hz (the amplitude was 50 mV) from room temperature to 60°C. For uncoated LLTO pellets, an Au/Pd layer was sputtered on both sides before the test. Coated LLTO pellets were tested directly without Au/Pd coating layer. The bulk ionic conductivity was calculated following the formula = σ L/RS, where R is the impedance for the fitted results in Nyquist plots, L is the ceramic thickness, and S is the effective surface area of the electrolyte. The galvanostatic charge/discharge characteristics for symmetric Li/Li cell were measured between -4.5 and 4.5 V with current densities of 0.01, 0.02 and 0.04 mA cm^−2^ at 60°C using a potentiostat (Biologic Sciences Instruments).

## Results and discussion

### Characterization of LLTO powders and pellets

LLTO powders exhibit different morphologies, as shown in .Supplementary Figure S1 The shape of the secondary particle for granular powder (G-LLTO) in Supplementary Figure S1A is spherical. For milled powder (M-LLTO in Supplementary Figure S1B), each secondary particle is more angular than G-LLTO. The secondary particles (with size of ∼40 μm) comprise of agglomerated primary particles with smaller sizes of ∼0.5 μm. Note that each secondary particle for M-LLTO appears to represent one primary particle. To enhance the sinterability of dense LLTO, G-LLTO and M-LLTO powders with various weight ratios are mixed and investigated. The optimization of the weight ratio between G-LLTO and M-LLTO powders is 70:30. While regarding to the mixed powder, each secondary particle contains larger primary LLTO particles surrounded by smaller primary particles. The secondary particle of mixed powder has the average particle size of ∼1 μ m as indicated in Supplementary Figure S1C.

The mixed LLTO electrolytes (70 wt% of G-LLTO and 30 wt% of M-LLTO) show the highest densification after sintering at 1,170°C for 12 h in air, compared with its single components as shown in Supplementary Figure S2. Furthermore, the SEM images for LLTO with the optimization of sintering temperatures is exhibited in Supplementary Figure S3. Green mixed pellets are sintered at 960°C, 1,050°C, and 1,170°C for 12 h in air atmosphere. Elevated sintering temperatures thus accelerate the growth of LLTO grains. The densification is enhanced with increased sintering temperature at the same time. As a result, the mixed LLTO pellets show void-free surface and indicate the highest densification after sintering at 1,170°C. However, the sintering temperature cannot be too high as Li would be lost during the sintering process (Li starts to be sublimated at temperatures above 900°C (Jonson and McGinn, 2018)).

**FIGURE 1:**
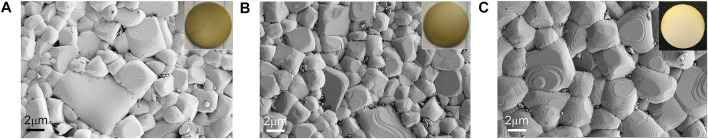
SEM pictures of sintered pellets (without polishing) at 1,170°C for 12 h in air: **(A)** G-LLTO; **(B)** M-LLTO; and **(C)** mixed LLTO.

In the insets of Figure 1, the sintered electrolytes have a light-yellow color on the surface. The surface of sintered LLTO pellets without any polishing is dense but relatively rough. The sintered pellets using G-LLTO and M-LLTO, respectively (in Figures 1A,B), indicate grains growth in different degrees. Most LLTO neighboring grains for mixed LLTO in Figure 1C have better contacts without significant porosity at 1,170°C. The dense microstructure of the LLTO electrolytes enables a homogeneous current distribution and prevents lithium dendrite penetration during the cycling process (Tsai et al., 2016).

**FIGURE 2:**
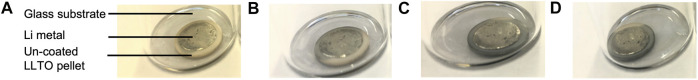
Photographs of LLTO contacts with Li-metal after: **(A)** 5 min; **(B)** 30 min; **(C)** 60 min; and **(D)** 90 min.

Figure 2 shows that the color of sintered LLTO pellets changes from white to dark gray and then to a deep black over time when bring in contact with Li metal. To avoid the exposure of Li metal to air, LLTO with Li-metal is sealed in glass containers filled with inert Argon atmosphere. It is clear that white LLTO is very sensitive to reduction and takes place in less than 5 min. Initially, white LLTO electrolytes would turn black after contacting with Li-metal after 90 min. The SEM micrograph in Supplementary Figure S4 shows that the surface of the white and black LLTO pellet is not flat. The roughness of both pellets depends on the cut-off procedure via low-speed diamond saw. This result supports that color change of LLTO has no apparent effect on the surface morphology of pellets.

**FIGURE 3:**
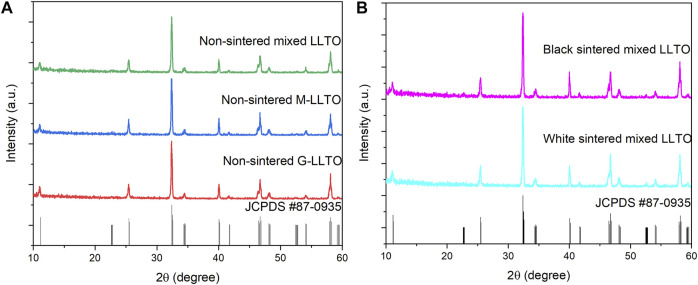
2D stacked XRD spectra of: **(A)** non-sintered LLTO pellets; and **(B)** sintered mixed LLTO pellets.

The XRD patterns of all pellets are plotted in Figure 3 and match to a perovskite ABO_3_ superstructure (tetragonal structure JCPDS #87-0935) (Yu et al., 2018; Li et al., 2019). Figure 3A proves that the bulk structure of white LLTO remains unchanged after mixing G-LLTO and M-LLTO powder. Figure 3B also validates that sintering at 1,170°C for 12 h in air does not affect tetragonal structure of black LLTO (when it is in contact with Li metal). Also, the LLTO structure would be changed from tetragonal to cubic after sintering over 1,500°C.

### Ionic conductivity of sintered LLTO pellets

**FIGURE 4:**
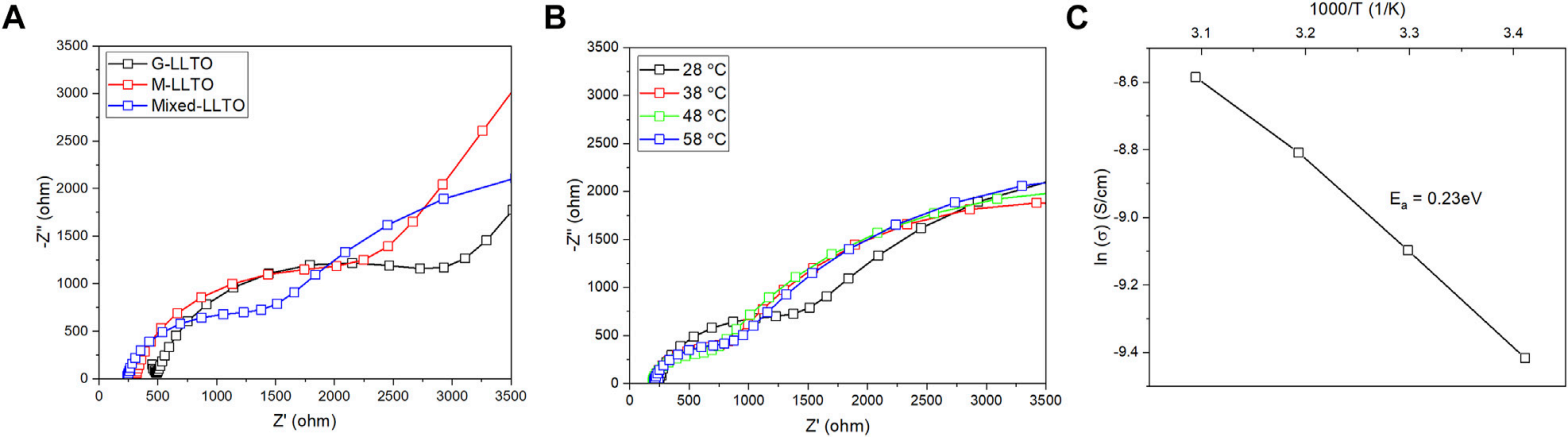
**(A)** AC impedance spectra of sintered un-coated LLTO pellets at room temperature; **(B)** Nyquist plot of sintered mixed LLTO pellet during 28°C–58°C; and **(C)** Arrhenius plot of sintered mixed LLTO pellet during 28°C–58°C.

Figure 4 compares EIS curves of sintered un-coated LLTO pellets. The resulting Nyquist plots contain one semicircle and an inclined straight line. In Figure 4A, the calculated bulk ionic conductivity of the G-LLTO pellet is $4.7\times 10^{-5}$ S cm^−1^ at room temperature. Because of the decreased particle size, the bulk ionic conductivity of M-LLTO electrolyte increases to $1.8\times 10^{-4}$ S cm^−1^. Mixed LLTO electrolyte presents the highest bulk conductivity of $2.1\times 10^{-4}$ S cm^−1^ at room temperature as calculated from Figure 4B. This enhancement might be attributed to the presence of smaller secondary particles within the grains of G-LLTO powder, which minimizes grain boundary resistance and increases the contact area between neighboring grains (Cheng et al., 2015). The ionic conductivity of the mixed electrolyte increases with temperature and follows an Arrhenius behavior as indicated in Figure 4C with an activation energy of 0.23 eV.

### Instability of LLTO electrolytes against Li-metal

**FIGURE 5:**
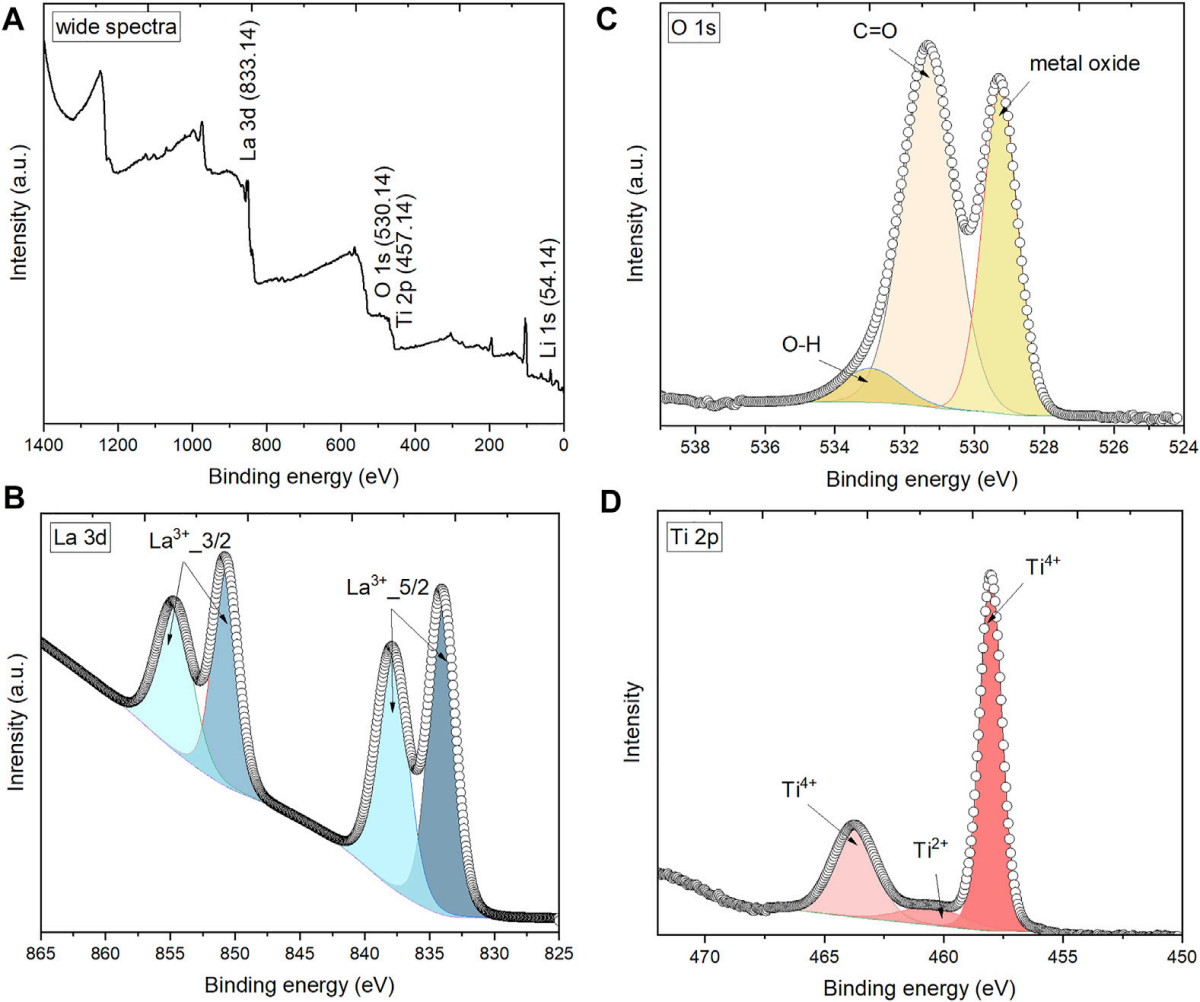
XPS spectra of black sintered LLTO pellet: **(A)** wide survey; **(B)** La 3d region; **(C)** O 1s region; and **(D)** Ti 2p region.

To further confirm the composition and chemical nature of black sintered LLTO pellets, XPS analysis is conducted on the collected black samples and the results are shown in Figure 5. The XPS spectra in Figure 5A consist of major peaks assigned to Li 1s, La 3 days, Ti 2p, O1 1s, and C 1s. C 1s peak at 284.8 eV is used as a reference peak for calibration. High-resolution peaks of La 3 days 3/2, La 3 days 5/2, and O 1s are shown in Figures 5B,C, respectively. In Figure 5D, the Ti 2p core spectrum reveals two main components: the peaks around 458.6 and 463.3 eV are associated with Ti^4+^ (Wagner et al., 2003; Biesinger et al., 2008). The atomic content of Ti^4+^ on the surface of the black sample is 84.48%. For black LLTO pellets, Ti^4+^ is heavily reduced to the lower valence of Ti after assembling with Li metal, corresponding to the peak around 460.5 eV that is associated with Ti^2+^ (Biesinger et al., 2008; Biesinger et al., 2011).

### Resistance of coated LLTO electrolytes

The two sides of sintered mixed LLTO pellets (non-polished) are coated with PEO-based gel membranes. The SEM images for the morphology of coated-LLTO with each interfacial layer are shown in Supplementary Figure S5. LLTO pellets modified by PEO-based gel films are smooth and uniform. PEO-based gel can be observed in the cracks and porosities of LLTO pellets and can thus provide effective pathways for Li-ion transportation even if the ceramic pellet cracks during cell assembly. High-resolution EDX mapping in insets of Supplementary Figure S5 exhibits a continuous and conformal coating of polymer on the surface of LLTO pellets, enabling effective protection of LLTO pellets from Ti ion reduction.

**FIGURE 6:**
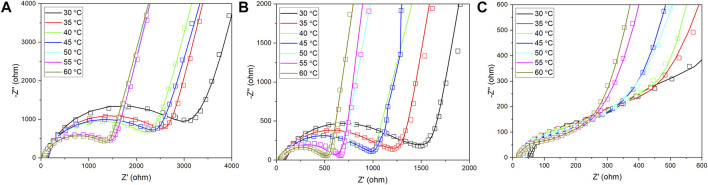
Nyquist plots of LLTO pellets during 30°C–60°C coated by: **(A)** PEO; **(B)** PEO-LiTFSI; and **(C)** PEO-LiTFSI-SN.

The AC impedance results of coated LLTO electrolytes during testing at 30°C-60°C are illustrated in Figure 6. The low-frequency semicircle in all curves is attributed to the total resistance of the coated electrolytes. Compared to the total resistance PEO-LLTO and PEO-LiTFSI (PL)-LLTO as shown in Figures 6A,B, the value of PEO-LiTFSI-SN (PLS)-LLTO in Figure 6C is the smallest (84.88 Ω cm^−2^ at 60°C) because the gel electrolyte has high ionic conduction compared with PEO or PL. Thus, it is verified that PLS with a weight ratio of 35%:30%:35% enables more lithium-ion pathways and enhances the polymer membrane’s ionic conductivity.

To determine the total activation energy for ion migration, temperature-dependent measurements are obtained at the same frequency range (1 MHz-0.01 Hz) by changing the temperature from 30°C to 60°C as plotted in Supplementary Figure S6. The calculated activation energy for each coated-LLTO is listed in Supplementary Table S1. PL-LLTO has the lowest activation energy of 14.85 kJ mol^−1^, as shown by the reduced interfacial resistance mentioned before. Compared to PEO only, the introduction of LiTFSI salt is helpful to reduce the activation energy and increase the ionic conductivity. For PLS-LLTO, it is obvious that after 55°C, the slope of the line decreased due to the reduction of activation energy. The slope change at higher temperatures is possibly due to “melting” phase transition of SN and PEO. Nevertheless, at low temperatures, the mobility of Li-ions is restricted (Polu et al., 2015). We assume that the increase in activation energy for PLS is due to the phase transition of SN to the plastic solid phase. As a result, PLS-LLTO increases the total resistance and brings on a higher energy barrier for lithium-ion transport. We would test PLS-LLTO in symmetric Li cells to further verify this hypothesis.

### Performance of symmetric Li | coated-LLTO | Li cell

Supplementary Figure S7 exhibits the color of coated LLTO pellets after cycling in symmetric cells. PEO membrane cannot protect LLTO well due to several darker grey dots (in Supplementary Figure S7A). The black circle in the middle of the P-LLTO and PL-LLTO (in Supplementary Figure S7B) pellets is Li foil. In contrast, Li foil is absent on the surface of PLS coated-LLTO as evidenced by Supplementary Figure S7C. It may be due to the softness change of the gel membranes at room temperature. The separation of Li foil from PLS coated-LLTO could also indicate increased interfacial resistance. Moreover, partial Ti reduction increases the total resistance of LLTO pellets. Compared to the ineffective coating of PEO, the color of PL or PLS coated-LLTO remains unchanged. Hence, it is proved that PL and PLS could protect LLTO against Li metal.

**FIGURE 7:**
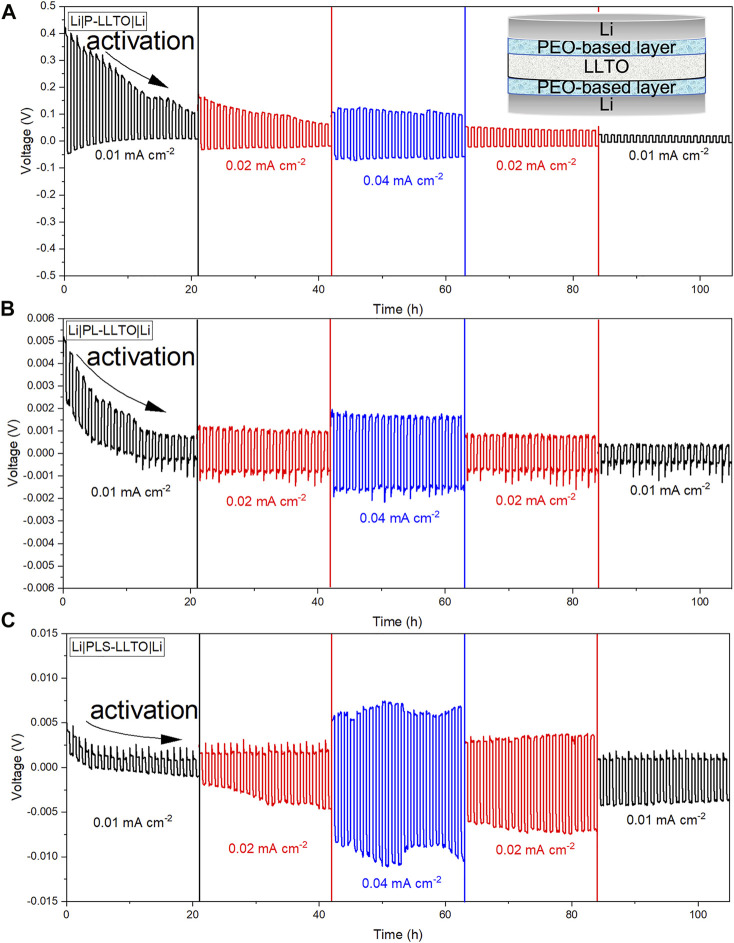
Cycling performance of symmetric Li cells at different current densities (tested at 60°C) and LLTO was coated by: **(A)** PEO; **(B)** PEO-LiTFSI; and **(C)** PEO-LiTFSI-SN.

Galvanostatic charge/discharge testing in symmetric Li cells (cell configuration is added in Figure 7 inset) are performed to evaluate the interfacial stability and voltage polarization at 60°C. The time-dependent voltage profile of the Li | gel-LLTO | Li cells are plotted and analyzed. LLTO coated by three gel membranes perform relatively stable plots without noise compared to un-coated LLTO as plotted in Supplementary Figure S8, as evidenced by which a better interfacial contact between coated-LLTO and Li. Besides, the flat voltage plateau with a small variation for three cells with coated-LLTO is support of the excellent interfacial stability (Chen et al., 2020). The open circuit voltage (OCV) is around 1.5 V and drops down to 0 V after resting for 12 h. To study the reason why the OCV is not 0 V, the assembled cell is rested for 12 and 24 h before cycling to investigate the difference. Voltage differences with the other cells studied here may be due to good Li-ion conduction of gel electrolytes. The interface activation process can be seen from the voltage profile of the stripping-plating cycle, where the voltage decreases in the first 20 h and then becomes relatively stable (Ulissi et al., 2016). The related tiny noise in the voltage profile is attributed to localized voids forming at the Li/coated layers interface as Li is stripped away from the anode; the voids likely formed because of insufficient pressure applied to the symmetric cell during cycling (Seitzman et al., 2018). Small asymmetry of the whole profile could be related to the still inefficient transition pathways and partial distortion between coating layers and LLTO (Chen et al., 2017).

Compared to the overpotential for un-coated LLTO at a current density of 0.04 mA cm^−2^, PEO coated-LLTO (P-LLTO) pellet has a larger value as represented in Figure 7A. This may be due to PEO degradation during the cycling at different current densities. Besides, as confirmed by the color change of PEO-LLTO pellet after cycling (in Supplementary Figure S7A) the reduction of LLTO might affect the interfacial stability against Li and increase the total cell resistance (Zhang et al., 2022). In comparison, the overpotential is only 1.7 mV at a current density of 0.04 mA cm^−2^ for PL-LLTO pellet as indicated in Figure 7B. The smallest value of overpotential with the stable trend and mitigated noise suggests enhanced interface stability and favored electrochemical reaction with Li-metal anode (Ulissi et al., 2016). The hysteresis change in voltage trace implies that the voltage shape after extended cycling cannot be fully captured by morphology-driven reaction kinetics (Chen et al., 2017). Regarding the reversible behavior of gel-LLTO for Li stripping and plating, the profile indicates stable performance when the current density is dropped down to the original value of 0.01 mA cm^−2^.

The larger overpotential value for PLS-LLTO pellet as shown in Figure 7C proves our assumption based on its higher activation energy during 30°C-60°C. PLS with SN brings on increased interfacial resistance against Li metal. This claim agrees with the observation for cycled PLS coated-LLTO (from opened symmetric Li cell in Supplementary Figure S7C) that Li metal is completely separated from PLS. Insufficient contact during the cycling causes polarization and more potential loss.

**FIGURE 8:**
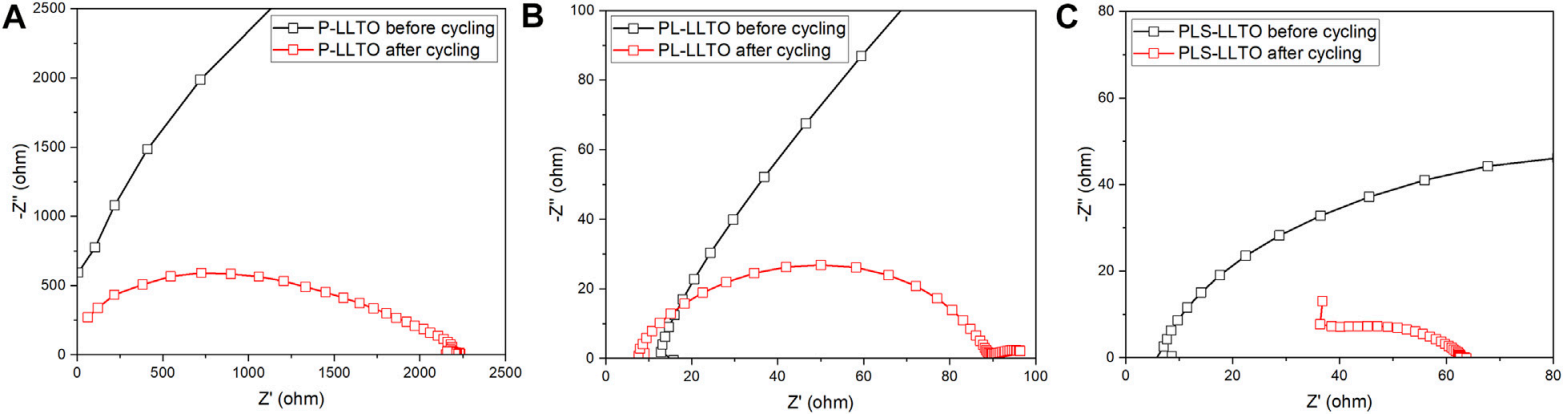
AC impedance symmetric Li | PEO-based coated-LLTO | Li cell before and after cycling (tested at room temperature): **(A)** PEO coated-LLTO; **(B)** PL coated-LLTO; and **(C)** PLS coated-LLTO.

To further examine the cycling stability of three PEO-based membranes on LLTO pellets, Figure 8 compares the resistance of symmetric Li cells before and after cycling at room temperature. The low-frequency semicircle corresponds to the total resistance of symmetric Li cells with coated-LLTO. Overall, there is a significant drop of total resistance for three gel coated-LLTO, proving polymer-based coatings’ effectiveness in improving interfacial contact. The large resistance of Li | PEO coated-LLTO | Li before galvanostatic cycling in Figure 8A might be ascribed to ineffectively interfacial contact between PEO and Li. PEO polymer is highly crystalline at room temperature, thus resulting in considerable impedance for Li^+^ transport (Lyu et al., 2020). During the cycling at 60°C, moderating the modulus of the soft-phase regions for PEO could be moderated, which helps to decrease the total resistance (Korley et al., 2006). However, the total resistance of the cell is still relatively high after cycling, which may be owing to PEO degradation (i.e., the change of crystallization conditions) (Scheirs et al., 1991) and its instability (i.e., the deterioration of physical properties) (Li D. et al., 2018B; Zhang et al., 2021) against Li. The total resistance for PL coated-LLTO (accessed from in Figure 8B) after cycling is subtly larger than PLS coated-LLTO (see in Figure 8C), possibly because the PLS membrane itself has lower resistance.

Table 2 and Supplementary Table S2 summarize the total polarization resistance and ionic conductivity of coated LLTO in symmetric Li cells. Stable potential is the average voltage window (the plus between positive potential and the absolute negative potential). The observed potential from all cells includes polarization losses from interface1 and ohmic losses from the LLTO pellet. The volume change of Li anode during cycling may be the origin of the overpotential change. The alignment of both Li foils may affect the polarization resistance with coating layers (Zhang et al., 2021). R_total_ (ohm) is calculated from ohm’s law: R = V/I, where V is the stable potential (mV), and I is the current density (mA cm^−2^). R_total_ contains R_bulk_ (the resistance of the LLTO pellet), R_interface1_ (the polarization resistance between PEO-based layers and Li metal), and R_interface2_ (the interfacial resistance between coated layers and LLTO pellet). R_1_ (ohm cm^−2^) is R_total_/surface area (cm^2^) and R_2_ (ohm cm^−1^) is R_total_/ thickness (cm).

**TABLE 2:** Summary of the total ionic conductivity for LLTO coated by three interfaces in symmetric Li cell (tested at 60°C, with decreased current density).

Interface	Current density (mA cm^−2^)	Stable potential (mV)	R_total_ (ohm)	R_1_(ohm cm^−2^)	R_2_(ohm cm^−1^)	$\sigma_{\mathrm{total}}$ (mS cm^−1^)
PEO	0.04	84.5	3,755.56	2,125.21	50,074.07	0.011
	0.02	32	2,844.45	1,609.63	37,925.93	0.015
	0.01	13.5	2,400	1,358.12	32,000	0.018
PL	0.04	1.55	68.89	38.98	810.46	0.70
	0.02	0.7	62.22	35.21	732.03	0.77
	0.01	0.35	62.22	35.21	732.03	0.77
PLS	0.04	7.25	322.22	182.34	3,790.85	0.15
	0.02	5.1	453.33	256.53	5,333.33	0.11
	0.01	2.35	417.78	236.41	4,915.03	0.12

During the cycling process, when the current density is decreased from 0.04 to 0.01 mA cm^−2^, the voltage profile of symmetric Li cells is relatively stable. For PL coated-LLTO, it is clearly observed that the total resistance (R_total_) almost remains unchanged with the decreasing current density. This represents that the applied current density of these three cells does not affect ionic conductivity for these three cells (Zhang et al., 2021). The smaller polarization voltage verifies the reduction of concentration polarization (Chen et al., 2017). Compared with PEO, LiTFSI in PEO builds more Li^+^ conductive pathways that promote rapid Li^+^ diffusion from the pellet to Li metal. PLS gel membranes show expansion during the stripping and plating process. This phenomenon is most likely the cause of the formation and disappearance of voids at the ceramic’s surface (Koshikawa et al., 2018). Produced voids or insufficient contacts would influence the value shift of the resistance. Among three gel membranes, PL has relatively stable resistance at different current densities, corresponding to a constant ionic conductivity of around 2 mS cm^−1^ at 60°C. The ionic conductivity of PLS-LLTO cell during the cycling is lower than PL-LLTO, which may be owing to phase transition of SN over 40°C.

Polymer gel membranes as interfaces could fulfill all voids on the surface and tend to infiltrate into the grain boundary regions of LLTO. Coating provides a relatively flat surface contact with Li metal and PL or PLS coated-LLTO could maintain the original white color after cycling. Furthermore, softer PL or PLS could effectively permeate into the cracks of LLTO pellets and prevent direct exposure of LLTO from Li-metal. Moreover, the total ionic conductivity of the LLTO pellet is improved by optimizing the conductivity of polymer membranes. For the PLS membrane itself, the addition of SN dissociates Li ions from LiTFSI and further maximizes the total ionic conductivity of LLTO pellets at 60°C. However, the large value of overpotential for PLS coated-LLTO in symmetric Li cells indicates that PLS has poor compatibility with Li metal that could be attributed to the poor chemical stability of nitriles towards chemical reduction by Li metal.

## Conclusion

In summary, we have successfully fabricated highly dense LLTO pellets via uniaxial cold press followed by sintering. EIS results from the Nyquist plot indicate that the sintered LLTO pellets have the largest bulk ionic conductivity of $2.1\times 10^{-4}$ S cm^−1^ at room temperature. In a highly dense LLTO system, M-LLTO powder with smaller secondary particles fills the voids between the primary particles in G-LLTO and enhances the densification of sintered LLTO pellets. Eventually, a denser LLTO can be beneficial to decrease the total resistance of electrolytes and thus increases the ionic conductivity.

Additionally, we have demonstrated that the instability of bare sintered LLTO electrolytes when in contact with Li metal (Ti^4+^ reduction), that is accompanied by visual color change, can be overcome by coating with an interfacial layer. Three types of polymeric interfacial layers have been investigated and their effects at the LLTO/Li metal interface have been deeply studied. Our study shows that PEO-LiTFSI (PL) and PEO-LiTFSI-SN (PLS) coated-pellets maintains original white color of LLTO after cycling with Li metal and effectively resolved the issue of LLTO chemical instability. PL or PLS gel membranes have a soft and flexible structure, which could fill all voids on the surface of LLTO and improve the interfacial contact with Li. In this way, protected LLTO shows chemical stability with Li-metal. They also facilitate the assembly of the fragile, thin ceramic electrolytes in cells. Among three types of gel membranes, LLTO coated by PLS gel shows the smallest total resistance of 84.88 Ω cm^−2^ and confirmed the excellent ionic conductivity of PLS gel. Nevertheless, the PLS membrane as the interface is less chemically stable in symmetric Li cell and brings on larger overpotential. Phase transition of SN at 60°C possibly increases the resistance at the PLS/Li metal interface and prohibits lithium ions diffusion. In contrast, PL coated-LLTO could decrease the energy barrier for Li^+^ transportation due to its better comparability with Li.

Our work proposes a novel microstructure of LLTO and hence enhances ceramic sinterability. Importantly, we have addressed the Ti^4+^ reduction issue when Li metal is in contact with LLTO and opened a new window for utilizing LLTO with Li-metal by introducing interfacial layers on ceramic electrolytes. Therefore, we believe that this approach sheds a light on the safe assembly of thin-film ceramic electrolytes in lithium metal battery.

## Data Availability

The raw data supporting the conclusions of this article will be made available by the authors, without undue reservation.
