# Supplementary material for: Engineered interfaces between perovskite La2/3xLi3xTiO3 electrolyte and Li metal for solid-state batteries
Source: Front Chem. 2022 Aug 10;10:966274. doi: 10.3389/fchem.2022.966274 (PMC9399616; doi:10.3389/fchem.2022.966274)
Supplement: Supplementary file 1 [file DataSheet1.docx]

Supplementary Material

# Supplementary Figures and Tables

## Supplementary Figures


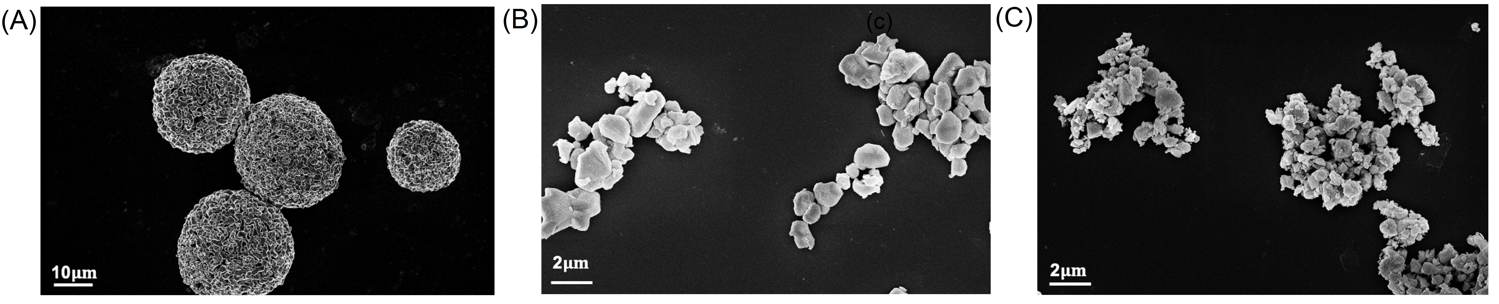


**Supplementary Figure 1.** SEM pictures of pristine LLTO powders: **(A)** granular LLTO; **(B)** milled LLTO; and **(C)** mixed LLTO


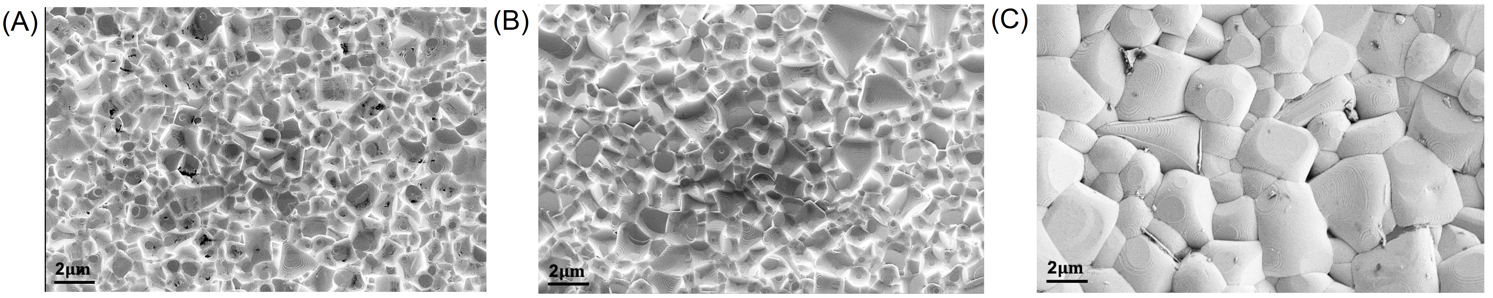


**Supplementary Figure 2.**  SEM pictures of the surface morphology for sintered mixed pellets with different weight ratio between G-LLTO and M-LLTO: **(A)** 60:40; **(B)** 65:35; and **(C)** 70:30


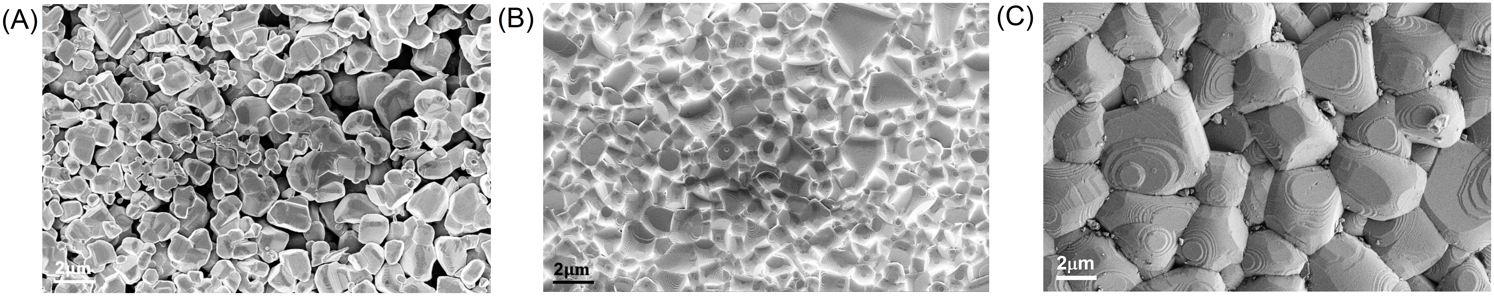


**Supplementary Figure 3.**  SEM pictures of the surface morphology for sintered mixed pellets in air at: (**A)** 960 ˚C; **(B)** 1050 ˚C; and **(C)** 1170 ˚C


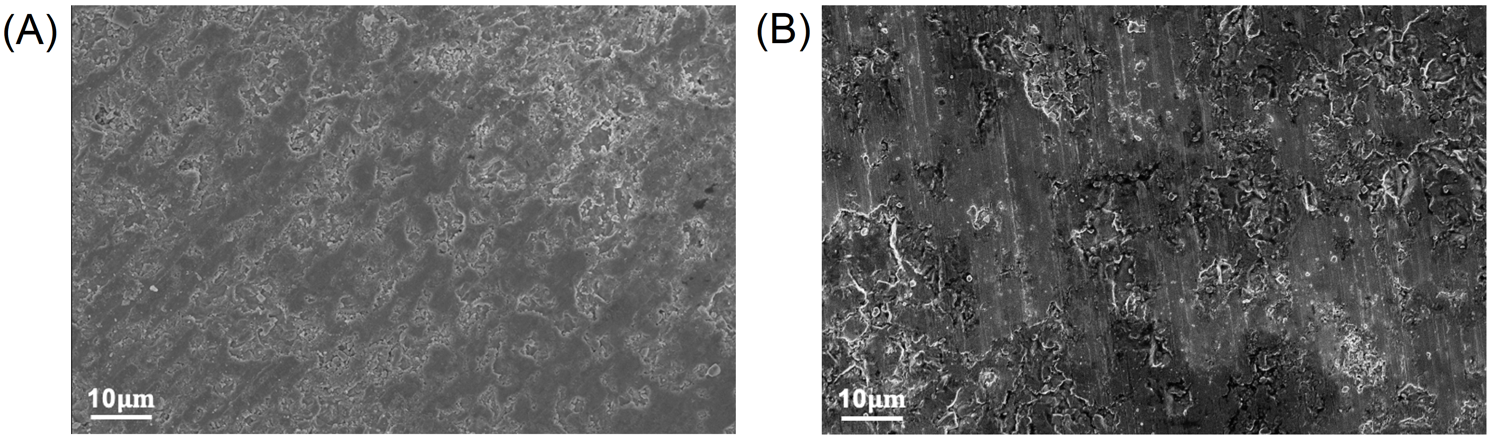


**Supplementary Figure 4.**  SEM pictures of cut-off sintered pellets: **(A)** white LLTO; and **(B)** black LLTO (color change after contacting with Li)


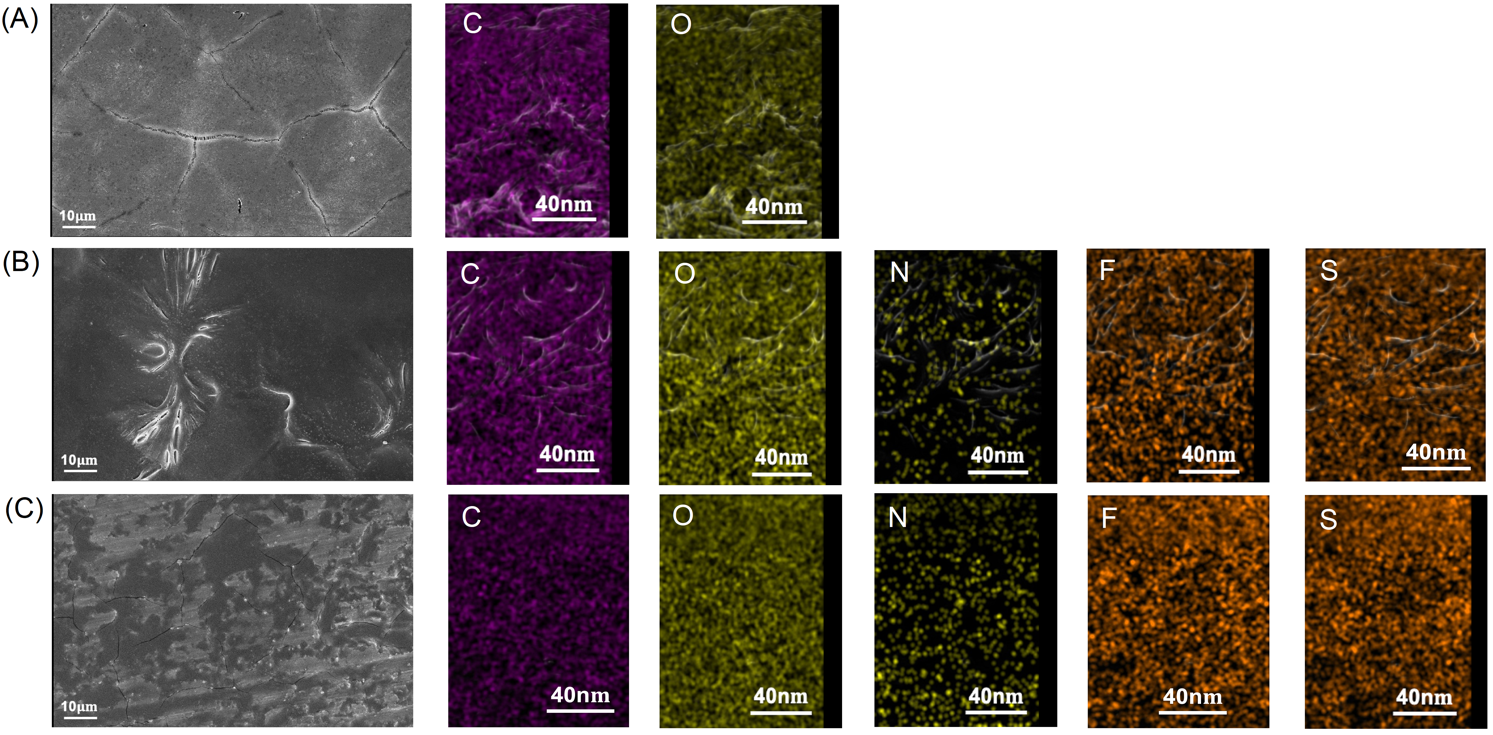


**Supplementary Figure 5.**  SEM pictures for the surface coated LLTO pellets by: **(A)** PEO; **(B)** PEO-LiTFSI; and **(C)** PEO-LiTFSI-SN and EDX mapping of C, O, N, F, S elements for each coated LLTO


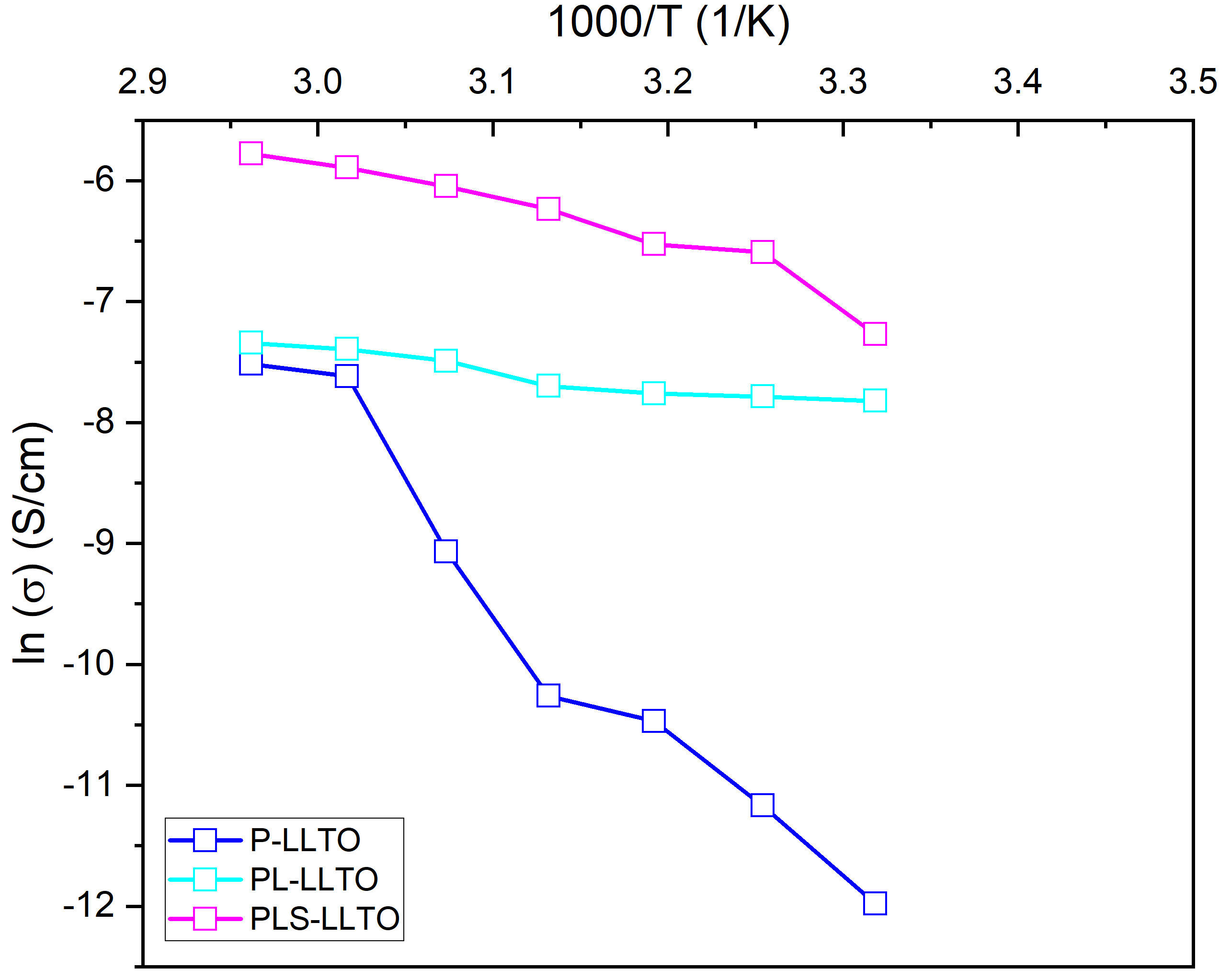


**Supplementary Figure 6.**  Arrhenius plots for coated pellet during 30 ˚C-60 ˚C


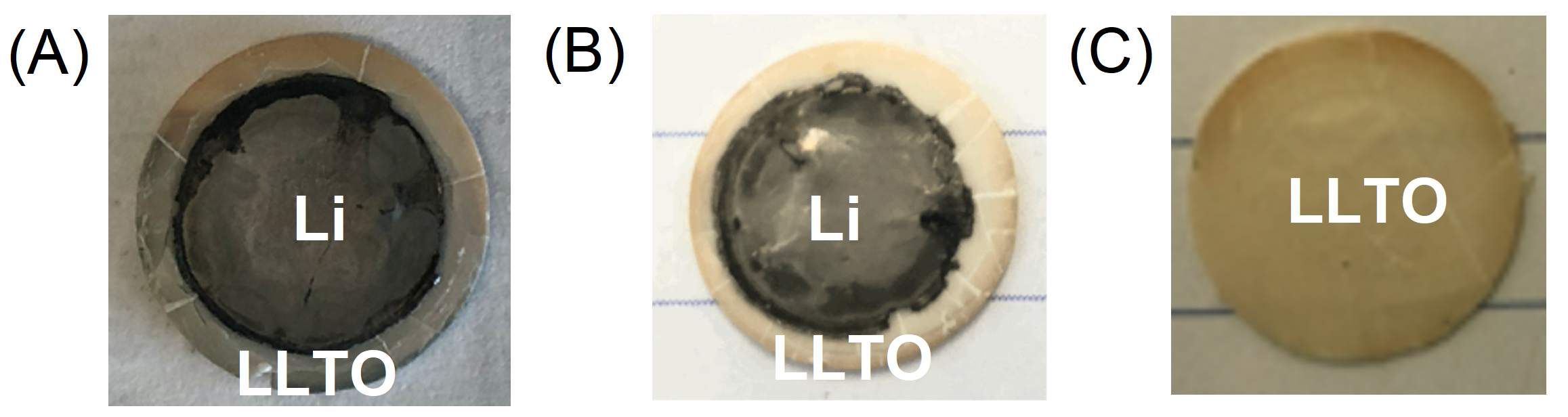


**Supplementary Figure 7.**  Photographs of LLTO pellets after cycling coated by: **(A)** PEO; **(B)** PEO-LiTFSI; and **(C)** PEO-LiTFSI-SN


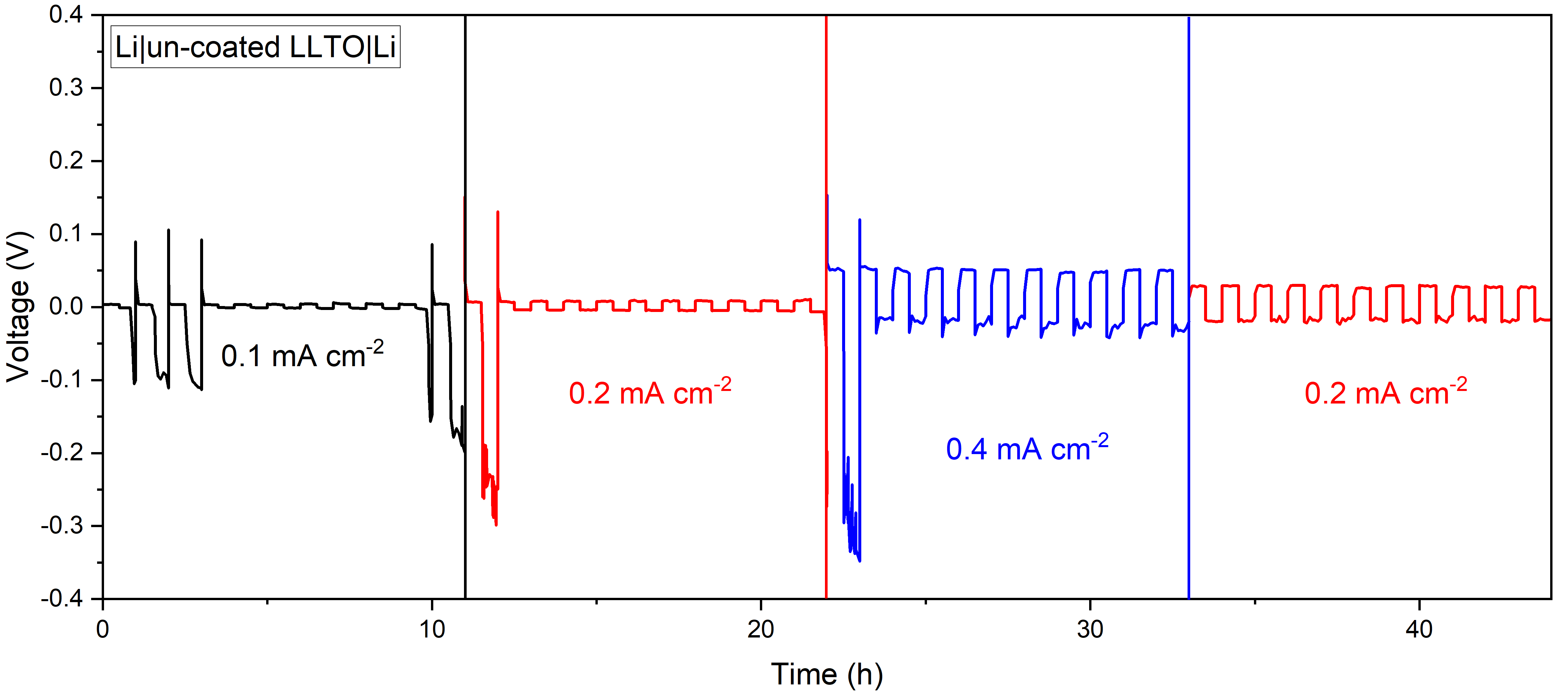


**Supplementary Figure 8.**  Cycling performance of symmetric Li | un-coated LLTO | Li cell at different current densities (tested at 60 ˚C)

## Supplementary Tables

**Supplementary Table 1.**  Summary of activation energy for coated-LLTO electrolytes

| Interfaces | Activation energy (kJ mol^-1^) |
| --- | --- |
| PEO | 131.06 |
| PL | 14.85 |
| PLS | 22.30 |

**Supplementary Table 2.**  Summary of the total ionic conductivity for LLTO coated by six interfaces in symmetric Li cell (tested at 60 ˚C, increased current density)

| Interface | Current density (mA cm^-2^) | | Stable potential (mV) | | R_total_ (ohm) | | R_1_(ohm cm^-2^) | | R_2_(ohm cm^-1^) | | $\boldsymbol{\sigma}_{\boldsymbol{total}}$(mS cm^-1^) | |
| --- | --- | --- | --- | --- | --- | --- | --- | --- | --- | --- | --- | --- |
| PEO | | 0.01 | | 50 | | 2829.42 | | 1601.13 | | 37725.62 | | 0.015 |
|  |  | 0.02 | | 46.85 | | 1325.58 | | 750.13 | | 17674.45 | | 0.032 |
|  |  | 0.04 | | 84.5 | | 1195.43 | | 676.48 | | 15939.07 | | 0.036 |
| PL | | 0.01 | | 0.45 | | 25.46 | | 14.41 | | 363.78 | | 1.89 |
|  |  | 0.02 | | 0.8 | | 22.64 | | 12.81 | | 323.36 | | 2.13 |
|  |  | 0.04 | | 1.55 | | 21.93 | | 12.41 | | 313.26 | | 2.19 |
| PLS | | 0.01 | | 0.7 | | 39.61 | | 22.42 | | 565.88 | | 1.21 |
|  |  | 0.02 | | 2.85 | | 80.64 | | 45.63 | | 1151.98 | | 0.60 |
|  |  | 0.04 | | 7.25 | | 102.57 | | 58.04 | | 1465.24 | | 0.47 |
